# The Prediction and Influential Factors of Violence in Male Schizophrenia Patients With Machine Learning Algorithms

**DOI:** 10.3389/fpsyt.2022.799899

**Published:** 2022-03-11

**Authors:** Tao Yu, Xulai Zhang, Xiuyan Liu, Chunyuan Xu, Chenchen Deng

**Affiliations:** ^1^Anhui Mental Health Center, Hefei Fourth People's Hospital, Affiliated Psychological Hospital of Anhui Medical University, Hefei, China; ^2^Anhui Province Maternity and Child Health Hospital, Hefei, China

**Keywords:** machine learning, violence, factor, schizophrenia, male

## Abstract

**Background:**

Early to identify male schizophrenia patients with violence is important for the performance of targeted measures and closer monitoring, but it is difficult to use conventional risk factors. This study is aimed to employ machine learning (ML) algorithms combined with routine data to predict violent behavior among male schizophrenia patients. Moreover, the identified best model might be utilized to calculate the probability of an individual committing violence.

**Method:**

We enrolled a total of 397 male schizophrenia patients and randomly stratified them into the training set and the testing set, in a 7:3 ratio. We used eight ML algorithms to develop the predictive models. The main variables as input features selected by the least absolute shrinkage and selection operator (LASSO) and logistic regression (LR) were integrated into prediction models for violence among male schizophrenia patients. In the training set, 10 × 10-fold cross-validation was conducted to adjust the parameters. In the testing set, we evaluated and compared the predictive performance of eight ML algorithms in terms of area under the curve (AUC) for the receiver operating characteristic curve.

**Result:**

Our results showed the prevalence of violence among male schizophrenia patients was 36.8%. The LASSO and LR identified main risk factors for violent behavior in patients with schizophrenia integrated into the predictive models, including lower education level [0.556 (0.378–0.816)], having cigarette smoking [2.121 (1.191–3.779)], higher positive syndrome [1.016 (1.002–1.031)] and higher social disability screening schedule (SDSS) [1.081 (1.026–1.139)]. The Neural Net (nnet) with an AUC of 0.6673 (0.5599–0.7748) had better prediction ability than that of other algorithms.

**Conclusion:**

ML algorithms are useful in early identifying male schizophrenia patients with violence and helping clinicians take preventive measures.

## Introduction

Patients with schizophrenia are more likely to exhibit violent behavior, compared with the general population. The relationship of schizophrenia and violent behavior has been repeatedly reported (1–3). Violent behaviors committed by patients with schizophrenia, not only cause physical damage or death to people, but also increase the social burden (4). Thereby early to recognize a minority of schizophrenia patients at an increased risk of violence may facilitate implementing violence prevention strategies and reducing damages caused by violent acts.

The risk assessment instruments for risk of violence in schizophrenia are widely applied in clinical practice. However, the available violence risk assessment tools rely on self-reported information, possess limited effective predictive power, and need mental health professionals' administer (5–7). Furthermore, patients often refuse to tell true ideas. The potential approach to identify an individual at risk of violence based on objective data is needed. Given only a minority of patients with schizophrenia possess violent tendencies, researchers have attempted to find factors that increase the risk of violent behaviors. There are many risk factors identified by conventional statistical methods (hypothesis testing) including abnormal brain cortical characteristics (8) substance use disorder (9) personality disorders (10, 11) and childhood victimization (12), but it is difficult to integrate these risk factors into a model to subsequently predict an individual's probability of committing violent behavior.

Machine learning as a comprehensive tool has several unique advantages in processing data and establishing models. ML can analyze a large of complex data simultaneously, incorporate different variables into the same model, select the optimal algorithm based on data structure, obtain the contribution of each variable to the constructed model (13).

So far, there have been two studies that employed ML algorithms to predict violent behavior in individuals with schizophrenia (14). In both studies, gender was included as a risk factor in the predictive models. However, some studies failed to find a link between gender and violent behavior in patients with schizophrenia (15). To control the influence of confounding gender factors, the predictive model established based on gender may be more suitable for clinical use. We utilized different ML algorithms combined with demographic characteristics, clinical and laboratory data to develop the models for predicting violence among male schizophrenia patients.

## Materials and Methods

### Participants

From March to August 2021, a total of 397 male patients with schizophrenia were recruited from the general psychiatry ward of the fourth people's hospital of Hefei. The proportion of doctors and the number of beds per ward were 56.00% and 90.9 in this hospital, respectively. The inclusion criteria were as follows: all patients who met schizophrenia diagnostic criteria in the Statistical Manual of Mental Disorders (DSM-V); patients who provided completed data. Exclusion criteria were as follows: patients with drug use disorders, patients with intellectual disability, patients with a diagnosis of organic mental disorder. All patients were divided into violence group and non-violence group according to whether they committed violent behavior within 1 month before admission to the hospital. Violent behavior was defined as an attempt or action to harm a target (16). Physical aggression against a person was determined as violent behavior in this study. The Ethics Committee of Anhui Medical University and Hefei Fourth People's Hospital approved the study.

### Materials

The demographic information sheet, the brief psychiatric rating scale (BPRS), the positive and negative symptom scale (PANSS), and the social disability screening schedule (SDSS) were adopted in our study. The self-developed demographic information sheet was used to obtain demographic characteristics (sex, age, education level, marital status, occupation, cigarette smoking, drinking and so on) and clinical data (the number of hospitalization, duration of disease, medication by drugs, family history, and so on). The severity of psychiatric symptoms in a patient was assessed by the BPRS, which consists of 18 items (17). The PANSS was used for assessing the symptom severity of patients suffering from schizophrenia (18). This scale includes positive syndrome, negative syndrome, and general psychopathology dimensions. There is a total of 30 items, with each item ranging from 0 to 7, and the higher scores indicate severer of the symptom. The social disability screening schedule (SDSS) with 10 items developed by the WHO Disability Assessment Schedule in 1988 was performed to measure a patient's social, occupational, and psychological functioning. The score of each item in this scale ranges from 0 to 2, with 0 indicating healthy or very minor defects, 1 indicating a functional obstacle and 2 indicating a serious function obstacle. According to cut-off scores, a total score ≥ 2 shows an obvious social function obstacle.

### Blood Samples

The fasting venous blood sample of each patient was collected on the morning of the second day after admission and subsequently was sent to the laboratory for testing. The biochemical indexes containing serum concentration of total cholesterol (TC), high-density lipoprotein (HDL), and triglycerides (TG), low-density lipoprotein (LDL), Apolipoproteins A-I (apo A-I), alkaline phosphatase (ALP), phosphorus (P), and glucose were measured by automatic biochemical instrument (Cobas c 702, Switzerland). Routine blood tests for white blood cell count (WBC), neutrophils (NEL), lymphocyte (Lym), monocyte (Mon), eosinophills (Eos), basophils (Bas), red blood cell (RBC), hemoglobin (HGB) were performed by an automatic blood analyzer (LH 750, USA). The serum concentrations of triiodothyronine (T3), thyroxine (T4), thyroid-stimulating hormone (TSH), free Triiodothyronine (FT3), and free thyroxine (FT4) were analyzed using an automatic electrochemiluminescence immunoassay analyzer (Cobas e 602, Switzerland).

### Prediction Model Development

Both LASSO and LR were employed to select these variables as input features in the prediction model. We then applied eight classification methods, including generalized linear models (glm), rpart, neural net (nnet), k-nearest neighbor (knn), random forest (rf), glmnet, support vector machine (svm), naive bayes (nb), and compared the ability to predict violent behavior among male schizophrenia patients.

Both glm and glmnet belong to linear regression, which is able to model the relationship between one or more independent variables and dependent variables by the least square function. The rpart belongs to a decision tree algorithm which works by splitting a data set into two parts recursively. For each step, considering the feature which results in the largest possible reduction in heterogeneity of the outcome variable, the segmentation is obtained. The nnet is consisted of a series of nodes in layers, where each node in one layer is connected to nodes in other layers. The knn, as the simplest classification algorithm, has been widely used in diverse fields. Where “K” represents the number of nearest neighbors, and the observations are classified into the category of the majority of “K” nearest neighbors. The results of the same data vary by selection of “K” values. The rf as “ensemble learning” can produce a single output or prediction by combining the results of multiple decision trees. The svm is an advanced algorithm which can deal with linear and non-linear data. It non-linearly assigns each feature regarded as a point to the multidimensional space, then finds an optimal plane and stratifies the two classes according to the maximum margin. The nb relies on Bayes'theorem, in which every feature of a class is assumed to be independent of each other. it can learn the prior knowledge of an event to generate the probability of the event occurrence.

### Prediction Model Evaluation

A total of 397 male patients were randomly divided into the train set and the test set in a 7:3 ratio, with the train set developing the models and the test set evaluating their performance. 10 × 10-fold cross-validation on the training set was utilized to tune parameters and counteract overfitting. The entire train set was equally divided into ten subsets. Each subset was served once as a validation set, and the remaining nine subsets were used for training the model. Subsequently, ten rounds of training and validation were conducted. After the cross-validation in the entire train set was finished, accuracy and kappa were used to generate the final model for each algorithm. For the final model for each algorithm, performance was evaluated using the testing set, in terms of AUC, balanced accuracy, kappa, sensitivity and specificity. The model with the highest AUC would be identified as the optimal model.

AUC refers to area under the receiver operator characteristic (ROC) curve, and combines sensitivity and specificity measures to describe algorithms' inherent validity. Accuracy is the proportion of correctly predicted patients from all patients. Sensitivity is the proportion of correctly predicted patients with violence among all patients with violence. Specificity is the proportion of correctly predicted non-violent patients among the non-violent population.

### Statistical Analysis

Statistical analysis was conducted in R software (version 3.6.2; The Comprehensive R Archive Network; http://cran.r-project.org). Continuous and categorical variables were expressed as mean ± standard deviation (SD) and proportions, respectively. *T-*test was conducted on the former and a chi-squared test on the latter. The packages for eight ML algorithms were the Caret packages, and glm, rpart, nnet, knn, rf, glmnet, svm, as well as nb were input, respectively. Both *P*-value and false discovery rate (FDR) were set at 0.05 (two-tailed), considered statistically significant.

## Results

### Demographic Characteristics

Of the 397 male schizophrenia patients recruited into this study, 36.8% (*n* = 146) had violent behaviors. The participants were 16–69 years old and their average age was 39.86 ± 13.98 years. The main demographic and clinical characteristics of patients with and without violence are showed in Table 1. There were significant differences in age, education level, duration of disease and positive syndrome between violent and non-violent groups. Whereas no significant differences were found in married statue, negative syndrome and BPRS between violent and non-violent groups.

**Table 1:** Main demographic and clinical characteristics of patients.

**Variable**	**Patients**	**Patients**	**Statistical**	***P* value**
	**with violence**	**without violence**	**value**	
	***N =* 146 (%)**	***N =* 251 (%)**		
Age	37.63 ± 12.56	41.16 ± 14.62	−2.543	0.011
Education level				
Primary school	29 (19.86)	35 (13.94)	8.979	0.011
Junior or senior	101 (69.18)	160 (63.75)		
high school				
College	16 (10.96)	56 (22.31)		
Married statue				
No single	24 (16.44)	49 (19.52)	0.585	0.444
Single	122 (83.56)	202 (80.48)		
Duration of disease	13.20 ± 9.47	15.54 ± 10.61	−2.271	0.024
Positive syndrome	27.14 ± 15.53	22.25 ± 15.26	3.058	0.002
Negative syndrome	44.58 ± 23.19	46.22 ± 22.72	−0.685	0.494
BPRS	31.63 ± 10.97	31.22 ± 28.68	0.532	0.867

### Identification of Risk Factors

**Figure 1:**
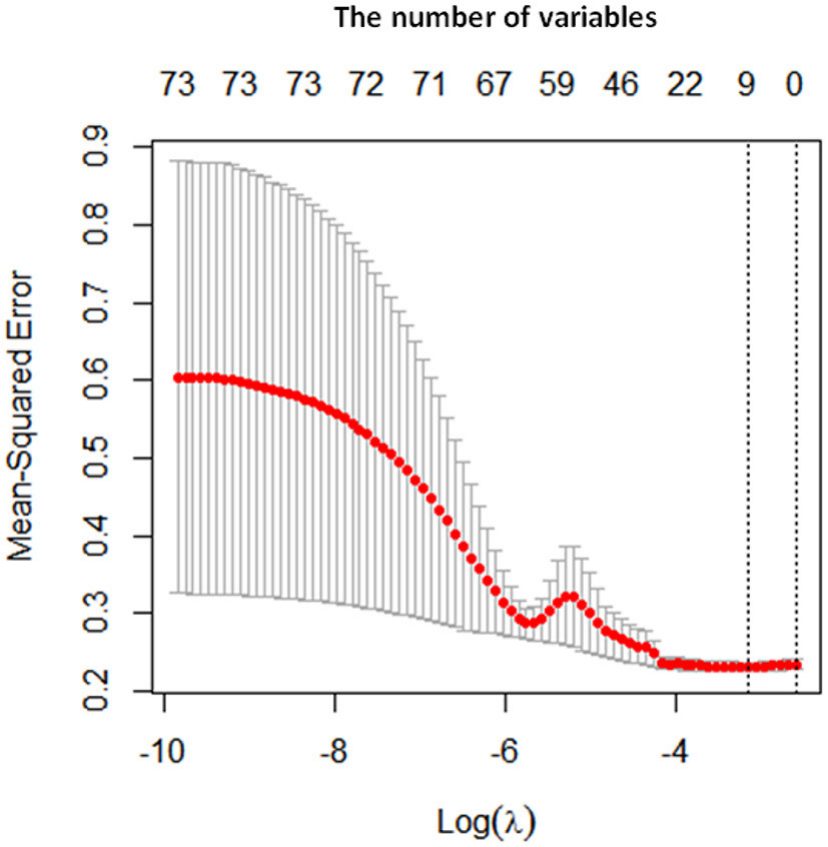
Prediction variables identified by LASSO. The x axis represents the log value of lambda, and y axis represents the mean squared error. The first dotted line represents the minimum mean squared error, corresponding to the optimum number of variables. The number at the top of the picture represents the number of variables.

Both LASSO and LR were conducted for selecting variables for model development. A total of 73 variables were enrolled into the variable shrinkage process, and eventually, 9 variables were determined by the LASSO, including age, education level, suicidal ideation, cigarette smoking, situation at birth, duration of disease, positive syndrome, SDSS score and uric acid showed in Figure 1 and Table 2. LR was then used to identify five factors including education level, suicidal ideation, cigarette smoking, positive syndrome, and SDSS score which were integrated into the predictive models, showed in Table 3.

**Table 2:** The variables selected by LASSO.

**Variable**	**Coefficient**
Age	−0.0031
Education level	−0.1198
Situation at birth	0.6040
Suicidal ideation	0.1864
Cigarette smoking	−0.1583
Duration of disease	−0.0035
Positive syndrome	0.0034
SDSS	0.0152
Uric acid	0.0003

**Table 3:** Independent factors associated with violence by logistic regression.

**Variables**		**β**	**SE**	**Wald**	***P* value**	**Adjusted *p* value**	**OR (95%CI)**
Education level		−0.587	0.196	8.961	0.003	0.015	0.556 (0.378–0.816)
Suicide ideation	yes	−1.063	0.539	3.892	0.049	0.049	0.345 (0.120–0.993)
	no	1					
Cigarette smoking	yes	0.752	0.295	6.517	0.011	0.018	2.121 (1.191–3.779)
	no	1					
Positive syndrome		0.016	0.007	4.886	0.027	0.034	1.016 (1.002–1.031)
SDSS		0.078	0.027	8.657	0.003	0.008	1.081 (1.026–1.139)

### Performance of Prediction Model

**Figure 2:**
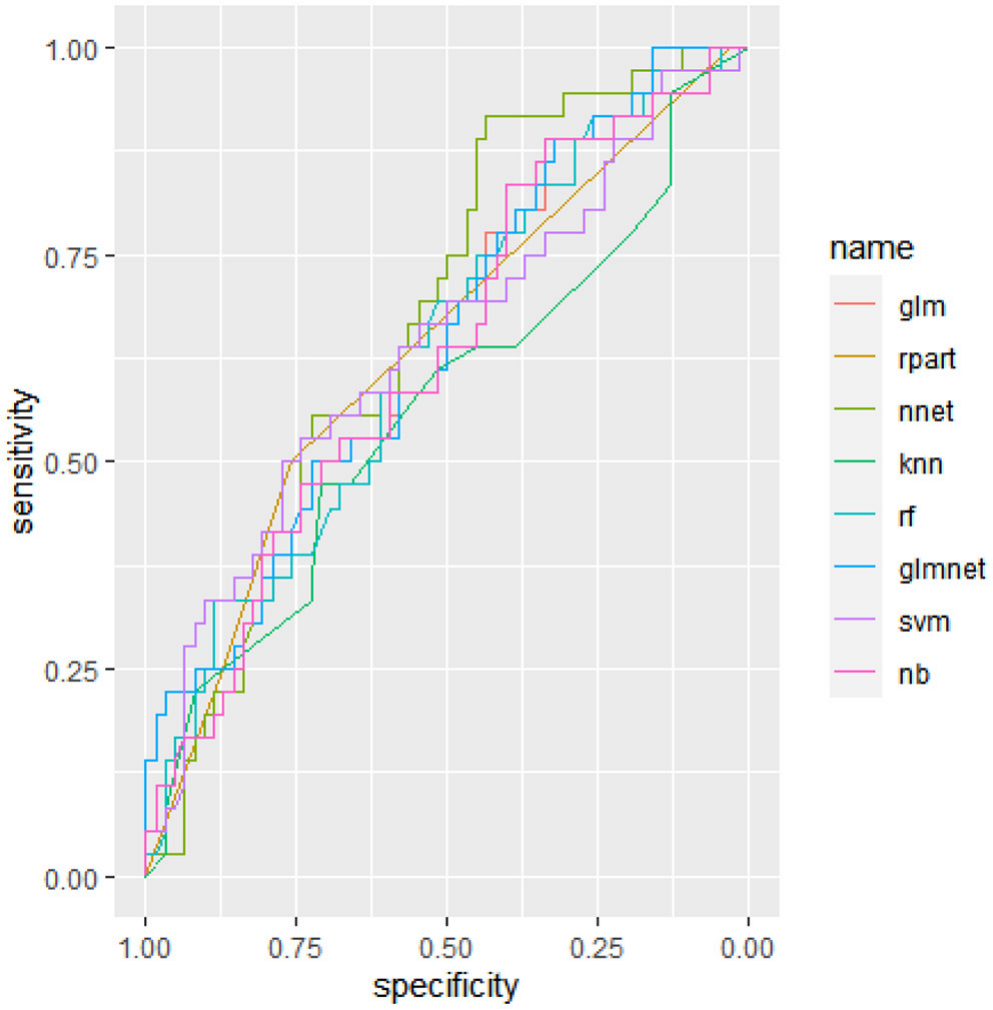
Comparison of performance of eight ML algorithms in testing set.

The performance of all algorithms in the testing set is showed in Table 4 and Figure 2. Based on five most important variables selected by LASSO and LR, the nnet had the highest predictive ability, with an AUC of 0.6673 (0.5599–0.7748).

**Table 4:** Prediction ability of ML algorithms in testing set.

**Algorithms**	**AUC**	**Balanced accuracy**	**Kappa**	**Sensitivity**	**Specificity**
glm	0.6454 (0.5327–0.7581)	0.5027	0.0061	0.1667	0.8387
rpart	0.6351 (0.5351–0.7350)	0.5757	0.1608	0.3611	0.7903
nnet	0.6673 (0.5599–0.7748)	0.6416	0.3007	0.4444	0.8387
knn	0.5661 (0.4436–0.6886)	0.7352	0.4934	0.5833	0.8871
rf	0.6353 (0.5218–0.7488)	0.7155	0.4605	0.5278	0.9032
glmnet	0.6449 (0.5323–0.7576)	0.5188	0.0432	0.1667	0.8710
svm	0.6400 (0.5223–0.7578)	0.5336	0.0826	0.0833	0.9839
nb	0.6288 (0.5143–0.7433)	0.5963	0.2152	0.3056	0.8871

## Discussion

The rate of violence in male participants was 36.8% in our study, which is consistent with the mean prevalence of aggressive behavior among 3,941 Chinese hospitalized patients with schizophrenia in 19 studies (19). In contrast, it is higher than the pooled prevalence of 3 to 15% in most Western countries (20). The discrepancy may be due to the fact that according to China's first mental health law implemented in 2013, psychiatric patients with an increased risk of violence have no right to decide whether to be admitted or not. Due to involuntary admissions, violent patients accounted for a relatively high proportion in our study.

Currently, the accuracy of existing violence risk assessment tools is low, and over 50% of patients at risk of violence are mistaken for those without violent risk (21). ML algorithms have been proved to be an effective method for predicting violent behavior among schizophrenia patients. For instance, Wang et al. utilized seven classification algorithms to predict violence status in schizophrenia individuals and found random forests showed better performance, its accuracy and AUC achieving 62% and 0.63, respectively (14). Another study determined gradient boosting as the best algorithm among seven algorithms, with its accuracy and AUC being 0.678 and 0.764 in predicting violent offending of forensic offender patients with schizophrenia, respectively (22). In this study, we conducted eight ML algorithms to differentiate violent and non-violent behaviors of male patients with schizophrenia. Through comparing with each other, the nnet had better performance, and its AUC of 0.6673 (0.5599-0.7748) was significantly better than chance. In terms of the ability to recognize male schizophrenia patients with violence, our model performance showed similar precision as was obtained in the previous studies (14, 22). Moreover, the nnet algorithm can calculate the probability of an individual committing violence. When a value exceeds 0.5, a patient might be considered to have a high risk of violence. Early detection of those helps to implement daily supervision, in order to urgently detect and handle violent behaviors. Furthermore, the integrated variables are generally common predictors for violence in schizophrenia, to some extent, supporting the temporal relationship of risk factors with violent behavior. It is important to note, despite the purpose of identifying patients at high risk of violence seems satisfactory, the optimal model identified in our study was unable to predict all patients at an increased risk of violence as an important limitation. Possible reasons are as follows. On the one hand, the exact mechanism of violent behavior in schizophrenia is still unknown. On the other hand, violence is caused by many factors, including genetic, social and environmental factors. Only some risk factors for violence were included in this study, so future studies should include more especial factors related to violence to improve the predictive accuracy. We also found the optimal algorithm varies between studies. The possible reasons for this difference are as follows: First, each algorithm has unique methodologies for processing data inputted and modeling complex relationships, and no single algorithm performs consistently best. Second, the performance of each algorithm is closely related to the type of data (23). Third, the method for cross-validation differed. our study adopted 10-fold cross-validation, Wang et al. (14) used 5-fold cross-validation, and Spnnweber et al. utilized a nested resampling approach.

The risk factors for violence among male schizophrenia patients incorporated into the predictive model included higher positive syndrome score, lower education level, suicide ideation, having cigarette smoking, and higher SDSS score. A higher positive syndrome score was observed in individuals who committed violent behavior, in comparison with those without violent behavior. This finding is consistent with previous research (24) suggesting schizophrenia individuals with violence had a more severe manifestation of the psychotic disorder. Positive symptoms, particularly delusions, hallucination, and persecutory ideation, accounted for a disproportionate amount of the violent behavior in schizophrenia (25, 26). It is suggested, more severe neurological dysfunction might result in the reduction of the capacity for timely behavioral modification or self-correction and the clinical effect of antipsychotic drugs (27, 28). We also observed lower education levels in male schizophrenia patients with violence, indicating the individuals with lower education levels tend to have severer psychopathological symptoms, which stop them from continuing learning (29). On the other hand, low education level is closely associated with low socioeconomic status, especially malnutrition and less cognitive stimulation, which might lead to cognitive function impairment (30). Patients who had suicidal ideation were less likely to commit violent behavior in our study, which is not consistent with findings from a meta-regression analysis of 110 studies showing suicidal ideation was not associated with violence risk (31). Despite the exact mechanism underlying the relationship between suicidal ideation and violence in schizophrenia is still unclear, we speculate individuals with suicidal ideation who always suffer from less severe psychiatric symptoms have the ability to suppress violent impluses (32). We found the cigarette smoking was associated with the increased risk of violence among male patients with schizophrenia. This finding is consistent with previous researches (33, 34). Cigarette smoking as a substance use, can promote the incidence of committing violence through the mediating role of psychiatric symptoms and social factors (35). Finally, Our findings also provided additional evidence that patients with higher SDSS scores tended to have more severe social functional impairment (22). Functional impairment is a recognized marker of illness severity, which is associated with the increased incidence of violence (36).

There were several limitations in this study. First, our study belongs to a cross-sectional design. Which does not confirm causation. Second, some important variables special to violence did not be considered in this study, such as childhood trauma, personality disorders, neuroimaging features. Future studies should include more variables to improve the predictive ability. Third, some information collected through self-report during treatment might be influenced by psychotic symptoms, leading to the limited reliability of the results. Fourth, our sample only enrolled male patients with schizophrenia from one hospital, and did not include patients living in the community, which limits the generalizability of the results. Future research should overcome these limitations to improve the prediction effect.

Regardless of the above-mentioned limitations, these are some strengths in our study. Considering the higher rate of violence in male patients and some risk factors special to gender, we developed prediction models for violence in male schizophrenia patients, which may be more suitable for clinical use. The sample in our study was relatively larger, increasing the credibility of findings in this study.

In conclusion, we found that the model developed by ML algorithms was useful in differentiating between patients with and without violent behavior. We also identified relevant risk factors associated with the occurrence of violence in schizophrenia, including education level, suicide ideation, cigarette smoking, positive syndrome, and SDSS. The probability of violent behavior committed by patients with schizophrenia can be calculated. For the individuals at high risk of displaying violent behavior, more timely, targeted, and effective measures are provided, in order to prevent violent behavior.

## Data Availability Statement

The raw data supporting the conclusions of this article will be made available by the authors, without undue reservation.

## Ethics Statement

Written informed consent was not obtained from the individual(s) for the publication of any potentially identifiable images or data included in this article.

## Author Contributions

TY designed the study, analyzed the data, and wrote the manuscript. XZ provided financial support. XL collected the relevant data. CX provided technological support. CD edited the manuscript. All authors contributed to the article and approved the submitted version.

## Funding

The study was funded by Hefei Fourth People's Hospital (HFSY202102) and Department of Science and Technology of Anhui Province (201904a0702009).

## Conflict of Interest

The authors declare that the research was conducted in the absence of any commercial or financial relationships that could be construed as a potential conflict of interest.

## Publisher's Note

All claims expressed in this article are solely those of the authors and do not necessarily represent those of their affiliated organizations, or those of the publisher, the editors and the reviewers. Any product that may be evaluated in this article, or claim that may be made by its manufacturer, is not guaranteed or endorsed by the publisher.
